# A Novel Assessment of Various Bio-Imaging Methods for Lung Tumor Detection and Treatment by using 4-D and 2-D CT Images

**Published:** 2013-06

**Authors:** Antony Judice A., Dr. K. Parimala Geetha

**Affiliations:** 1 Research scholar, Anna University, Chennai;; 2 Department of ECE, PJCE, India

**Keywords:** HMM, Segmentation, Feature Extraction, Baum-Welch Algorithm

## Abstract

Lung Cancer is known as one of the most difficult cancer to cure, and the number of deaths that it causes generally increasing. A detection of the Lung Cancer in its early stage can be helpful for Medical treatment to limit the danger, but it is a challenging problem due to Cancer cell structure. Interpretation of Medical image is often difficult and time consuming, even for the experienced Physicians. The aid of image analysis Based on machine learning can make this process easier. This paper describes fully Automatic Decision Support system for Lung Cancer diagnostic from CT Lung images. Most traditional medical diagnosis systems are founded on huge quantity of training data and takes long processing time. However, on the occasion that very little volume of data is available, the traditional diagnosis systems derive defects such as larger error, Time complexity. Focused on the solution to this problem, a Medical Diagnosis System based on Hidden Markov Model (HMM) is presented. In this paper we describe a pre-processing stage involving some Noise removal techniques help to solve this problem, we preprocess an images (by Mean Error Square Filtering and Histogram analysis)obtained after scanning the Lung CT images. Secondly separate the lung areas from an image by a segmentation process (by Thresholding and region growing techniques). Finally we developed HMM for the classification of Cancer Nodule. Results are checked for 2D and 4D CT images. This automation process reduces the time complexity and increases the diagnosis confidence.

## INTRODUCTION

Lung Cancer is the uncontrolled growth of abnormal cells that start off in one or both lungs; usually in the cells that line the air passages. The abnormal cells do not develop into healthy lung tissue, they divide rapidly and form tumors. As tumors become larger and more numerous, they undermine the lung’s ability to provide the bloodstream with oxygen. Tumors that remain in one place and do not appear to spread are known as “benign tumors”. Malignant tumors, the more dangerous ones, spread to other parts of the body either through the bloodstream or the lymphatic system. Metastasis refers to cancer spreading beyond its site of origin to other parts of the body. When cancer spreads it is much harder to treat successfully. Primary Lung Cancer originates in the lungs, while secondary Lung Cancer starts somewhere else in the body, metastasizes, and reaches the lungs. They are considered different types of cancers and are not treated in the same way.

The National Cancer Registry Programme of the Indian Council of Medical Research, which collected data from six different parts of the country, both rural and urban areas, showed varying figures in different area (3). While cancer of the trachea, bronchus and lungs was the most common form of malignancy in males in 1989 from Bombay, Delhi, and Bhopal this all are the major cities of INDIA. According to the National Cancer Institute, by the end of 2012 there will have been 226,160 new Lung Cancer diagnoses and 160,340 lung-cancer related deaths in the USA (1). According to the World Health Organization (WHO) (2), 7.6 million deaths globally each year are caused by cancer; cancer represents 13% of all global deaths. As seen below, Lung Cancer is by far the number one cancer killer. Total deaths worldwide caused by cancer each year: Lung Cancer - 1,370,000 deaths, Stomach cancer - 736,000 deaths, Liver cancer - 695,000 deaths, Breast cancer - 458,000 deaths.

The American Cancer Society says that Lung Cancer makes up 14% of all newly diagnosed cancers in the USA today. It adds that annually, more patients die from Lung Cancer alone than prostate, breast and colon cancers combined (in the USA). Lung Cancer can be broadly classified into two main types are small cell Lung Cancer and non-small cell Lung Cancer. These types are diagnosed based on how the cells look under a microscope.

Estimated new cases and deaths from Lung Cancer (non-small cell and small cell combined) in the United States in 2012: New cases: 226,160, Deaths: 160,340. Some of the CAD system uses SVM, Fuzzy Logic, Neural network algorithm. Their disadvantages are time consumption and needed a lot of data for training. So the Hidden Markov model is introduced for getting more advantage.

## PROPOSED METHOD

### CT scan

**Figure 1:**
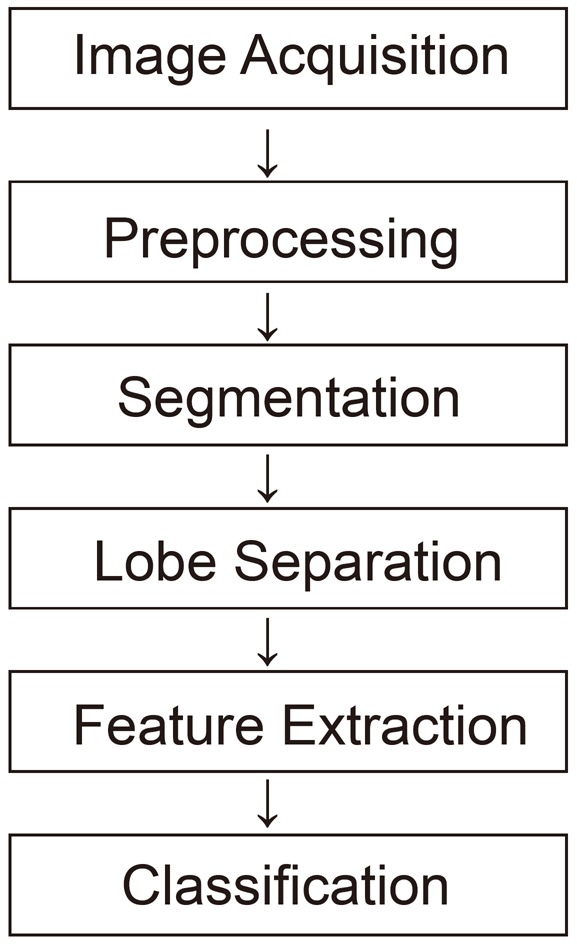
Flow of Bio-Imaging.

A CT scan stands for Computed Tomography scan. It is a painless, noninvasive test. It creates precise pictures of the structures in our chest, such as lungs. “Noninvasive” means that no surgery is done and no instruments are inserted into your body. It is also known as a CAT (Computer Axial Tomography) scans. It is a medical imaging method that employs tomography (4). Tomography is the process of generating a two-dimensional image of a slice or section through a 3-dimensional object (a tomogram) (Figure 1).

**Figure 2:**
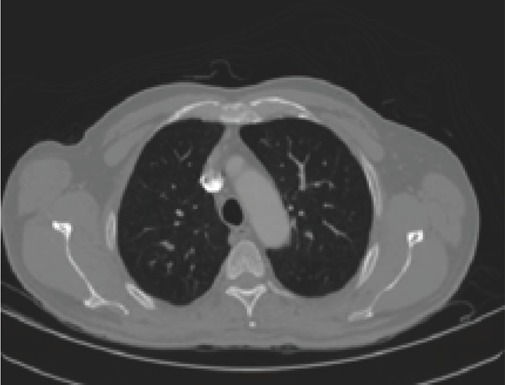
2-D CT Image.

A CT scanner emits a series of narrow beams through the human body as it moves through an arc, unlike an X-ray machine which sends just one radiation beam. Inside the CT scanner there is an X-ray detector which can see hundreds of different levels of density. It can see tissues inside a solid organ. This data is transmitted to a computer, which builds up a 3-D cross-sectional picture of the part of the body and displays it on the screen (Figure 2).

**Figure 3:**
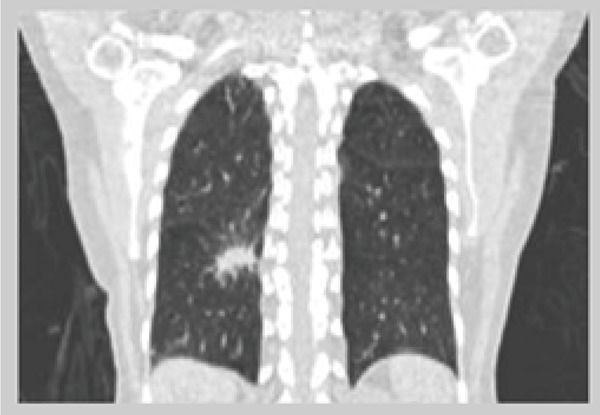
4-D CT Image.

In this paper we also discuss about the 4D CT images, 4D CT is a dynamic volume imaging system of moving organs with an image quality comparable to conventional CT. Dynamic cone-beam CT can realize it with several breakthroughs. They are: 1) large-area 2-dimensional (2D) detector; 2) high-speed data transfer system; 3) reconstruction algorithm; 4) ultra-high-speed reconstruction computer and 5) high-speed and continuous rotating gantry (5) (Figure 3).

4D CT images over-sampling images at every position of interest along the patient’s long axis. Each image is tagged with breathing signals and images are sorted retrospectively based on the corresponding breathing signals.

### Preprocessing

Preprocessing is very important in isotropic CT Lung images. Since isotropic CT images contain more noise than their clinical counterparts, that can be removed by Wiener filter. After removing the noise analyzing the histogram of the original CT image.

The most important technique for removal of blur in images due to linear motion or unfocussed optics and also due to vibrations. From a signal processing standpoint, blurring mainly occur due to poor sampling. Each pixel in a digital representation of the photograph should represent the intensity of a single stationary point in front of the camera. Unfortunately, if the shutter speed is too slow and the camera is in motion, a given pixel will be different of intensities from points along the line of the camera’s motion. This is a two-dimensional analogy to

G(u,v)=F(u,v).H(u,v)

where F is the Fourier transform of an “ideal” version of a given image, and H is the blurring function.

In the real world, however, there are two problems with this method. First, H is not known precisely. Engineers can guess at the blurring function for a given circumstance, but determination of a good blurring function requires lots of trial and error. Second, inverse filtering fails in some circumstances because the sinc function goes to 0 at some values of x and y. Real pictures contain noise which becomes amplified to the point of destroying all attempts at reconstruction of a Fest.

The best method to solve the second problem is to use Wiener filtering. This tool solves an estimate for F according to the following equation:

F^(u,v)=|H(u,v)|^2.G(u,v)/(|H(u,v)|^2.H(u,v)+K(u,v))

**Figure 4:**
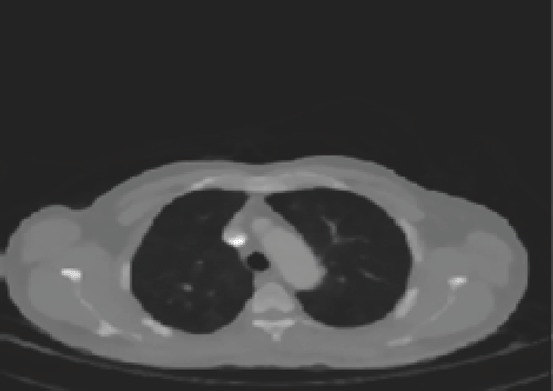
Filtered 2-D CT Image.

**Figure 5:**
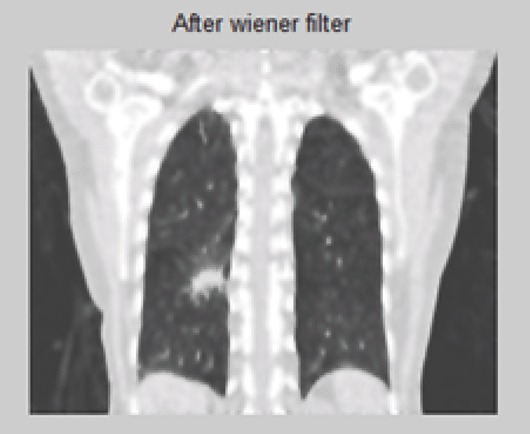
4-D Filtered Image.

K is a constant chosen to optimize the estimate. This equation is derived from a least squares method (Figure 4 and Figure 5).

**Figure 6:**
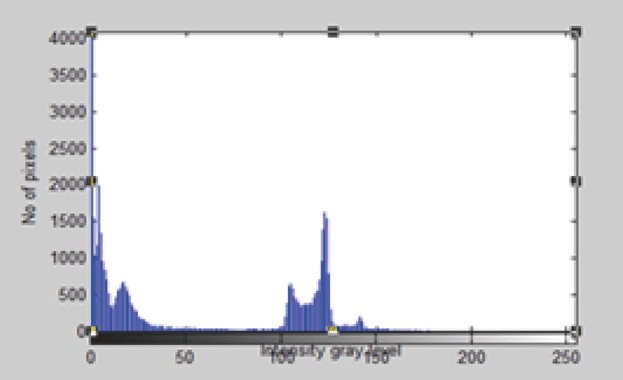
Histogram of the original 2-D CT Image.

**Figure 7:**
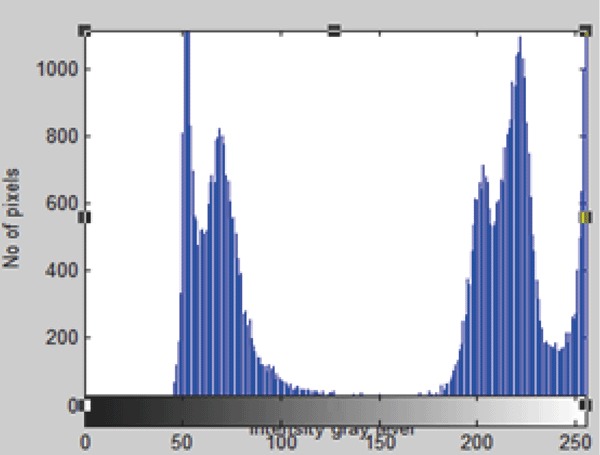
Histogram of the original 4-D CT Image.

A useful approach to this filter is optimization problem it minimize the mean-square value of the error signal that is defined as the difference between some desired response and the actual filter output. For stationary inputs, the resulting solution is commonly known as the Weiner filter (Figure 6 and Figure 7).

### Discrete Wavelet Transform

In numerical analysis and functional analysis, a discrete wavelet transform (DWT) is any wavelet transform for which the wavelets are discretely sampled. As with other wavelet transforms, a key advantage it has over Fourier transforms is temporal resolution: it captures both frequency and location information (location in time).The wavelet transform was borne out of a need for further developments from Fourier transforms. Wavelets transform signals in the time domain (rather, assumed to be in the time domain) to a joint time-frequency domain. The main weakness that was found in Fourier transforms was their lack of localized support, which made them susceptible to Heisenberg’s Uncertainty principle. In short, this means that we could get information about the frequencies present in a signal, but not where and when the frequencies occurred. Wavelets, on the other hand, are not anywhere as subject to it.

In our paper we discuss the Discrete wavelet transform as an efficient technique to find the noise reduction.

1. DWT transforms a discrete time signal to a discrete wavelet representation.
2. Provides better identification of data which is relevant to human perception.
3. It requires a significant amount of computation time.

**Figure 8:**
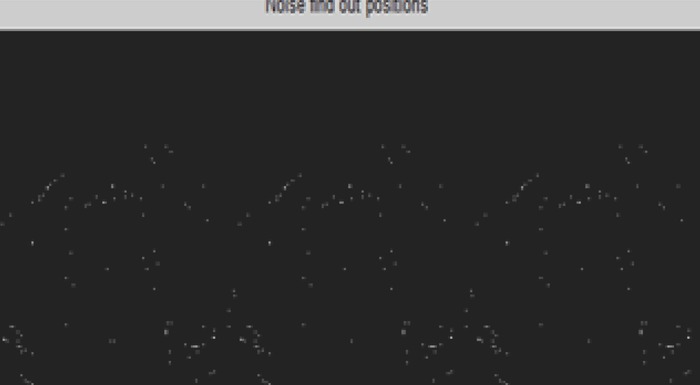
Noise find out position of 2-D CT image.

The figure illustrate the noise find out position of two dimensional CT image using Discrete wavelet transform (Figure 8).

### Segmentation

Image segmentation is an important concept in image processing. Segmentation is a process of extracting and representing information from an image is to group pixels together into regions of similarity. Segmentation of medical image of soft tissue into region is a difficult problem because of the large variety of their characteristics (7). Some of the segmentation methods are Thresholding, region growing, edge detection, ridge detection, morphological operations, fitting of geometrical models or functions and dynamic programming (6). In this technique we uses Thresholding and region growing. The goal of two methods is to identify the region of interest (ROI) which help in determining the cancer region. Classifiers are various types of neural networks, or Markov random field modeling, trained with a variety of local features including intensity, location, and texture measures (7). CADs can be divided into two groups (8): density-based and model-based approaches. In density-based detection methods employ techniques such as multiple Thresholding, region-growing, locally adaptive Thresholding in combination with region growing.

**Figure 9:**
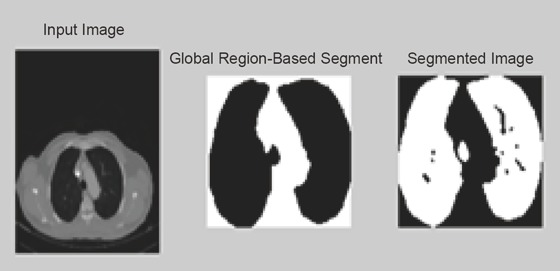
Segmented 2-D CT image using Region Growing.

**Figure 10:**
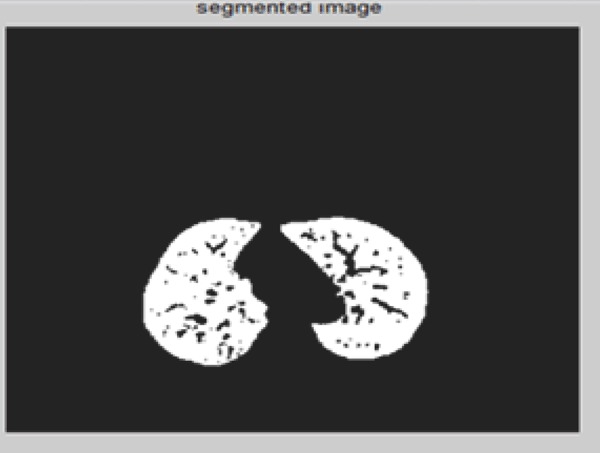
Segmentation using Adaptive Thresholding.

In this technique we uses global threshold, in global Thresholding the lung regions are separated by setting the threshold value (Figure 9 and Figure 10).

**Figure 11:**
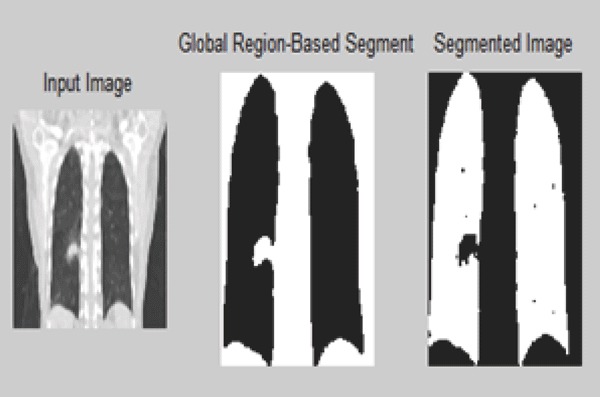
Segmented 4-D CT image using Region Growing.

**Figure 12:**
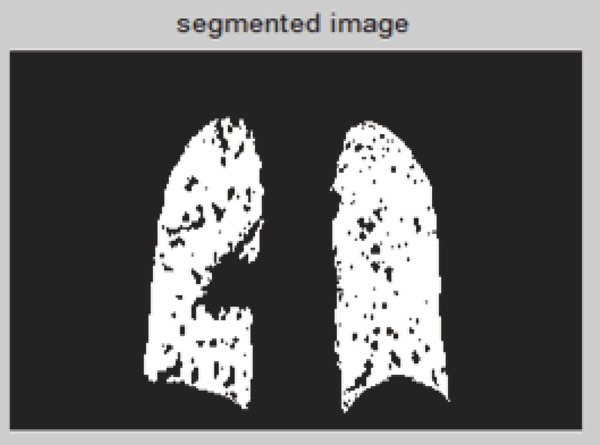
Thresholding approach of 4D CT image.

Figure segmentation using Thresholding illustrates the global Thresholding approach with threshold values 0.05555. Similarly using this approach left and right lung region are separated by setting different threshold values. Similarly for the 4-D images are illustrated in the below figure (Figure 11 and Figure 12).

Here threshold value used is 0.0678 for 4-D CT image.

### Lobe Separation

The goal of the lobe separation step is to completely separate the right and left lungs. Using a technique similar to that employed in dynamic programming is applied to find the maximum cost path through a graph with weights proportional to pixel gray-level (11). However, use a different strategy to find the dynamic programming search regions. In this method, a search region is found on a 2-D slice to successive slices. Because of the smooth pulmonary anatomy, the junction line position varies slowly through the data set.

To further reduce computation time, only apply the lung separation step to those slices that contain a single, large, connected lung component. A conditional dilation is then used to restore the approximate original boundary shape, without reconnecting the two lungs again.

The lobe segmentation algorithm works well for the automatic detection of the fissure locations and curvatures for both left and right oblique fissures (10).

### Feature Extraction

Features are functions of the original measurement variables that are useful for classification and/or pattern recognition. Feature extraction is the process of defining a set of features, or image characteristics, which will most efficiently or meaningfully represent the information that is important for analysis and classification. In Features extraction, extraction characteristics of the objects of interest, if selected carefully are representative of the maximum relevant information that the image has to offer for a complete characterization a lesion. Feature extraction methodologies analyze objects and images to extract the most prominent features that are representative of the various classes of objects. Features are used as inputs to classifiers that assign them to the class that they represent. Analysis with a large number of variables generally requires a large amount of memory and computation power or a classification algorithm which over fits the training sample and generalizes poorly to new samples. Feature extraction is a general term for methods of constructing combinations of the variables to get around these problems while still describing the data with sufficient accuracy. Edge detection, Corner detection, Blob detection, Ridge detection Shape Based Thresholding, Blob extraction, Template matching, Hough transform etc.

## CLASSIFICATION ALGORITHM

A random sequence has the Markov property if its distribution is determined solely by its current state. Any random process having this property is called a Markov random process. For observable state sequences (state is known from data), this leads to a Markov chain model. For non-observable states, this leads to a Hidden Markov Model (HMM).A hidden Markov model (HMM) is a statistical Markov model in which the system being modeled is assumed to be a Markov process with unobserved (hidden) states. An HMM can be considered as the simplest dynamic Bayesian network.

HMMs are composed of states, which are traversed according to transition probabilities. The sequence data is viewed as a series of observations emitted by the states, where an emission distribution over observations is associated with each state (12). Formally, an HMM is characterized by three stochastic matrices, called the initial, transition and observation matrices. The transition matrix, A, is a square matrix that holds the probabilities of transitioning from each state to any other.

The probability of transitioning from state i to state j is denoted by a_ij_. The initial distribution vector, π, is a column vector that stores the probabilities of starting in each state at the beginning of the sequence. π_i_ denotes the probability of starting in state i. Finally, the observation matrix, B, defines the probabilities of observing each base pair for every state. The probability of observing observation k in state j is denoted by b_j_(k).

**Figure 13:**
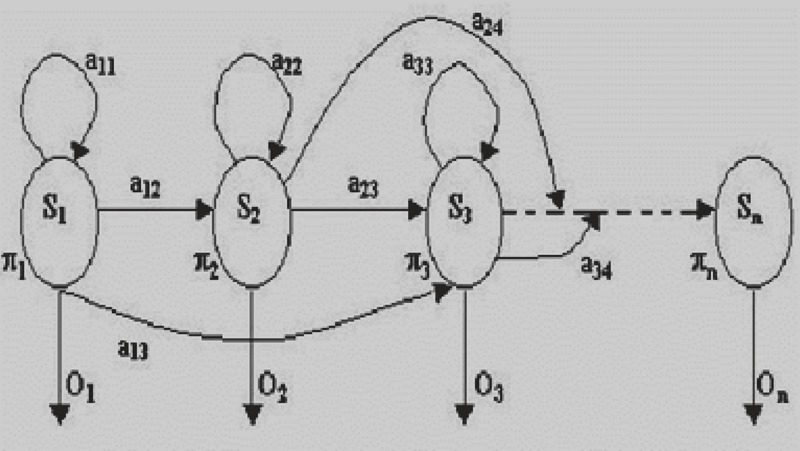
Example of a Hidden Markov Model. O_i_, initial state probabilities; a_ij_, state transition probability; b_j_(k) = probability of recognizing kith symbol in transition from i to j.

In a regular Markov model, the state is directly visible to the observer, and therefore the state transition probabilities are the only parameters. In a hidden Markov model, the state is not directly visible, but output, dependent on the state, is visible. Each state has a probability distribution over the possible output tokens. Therefore the sequence of tokens generated by an HMM gives some information about the sequence of states (13, 14). Note that the adjective ‘hidden’ refers to the state sequence through which the model passes, not to the parameters of the model; even if the model parameters are known exactly, the model is still hidden (Figure 13).

Most selected Training algorithm used is the Baum-Welch re-estimation Formulas. The Hidden Markov Model is a finite set of states, each of which is associated with a (generally multidimensional) probability distribution. So according to the training of HMM with regarding the features it will reports the accrue out more effectively (14). An artificial neural network is a structure which will attempt to find a relationship i.e. a function between the inputs, and the provided output(s), in order that when the net be provided with unseen inputs, and according with the recorded internal data (named “weights”), will try to find a correct answer for the new inputs.

The main difference could be this: In order to use a Markov chain, the process must depend only in its last state. For use a neural network, you need a lot of past data.

The Baum-Welch algorithm can be used to train an HMM to model a set of sequence data. The algorithm starts with an initial model and iteratively updates it until convergence. The algorithm is guaranteed to converge to an HMM that locally maximizes the likelihood (the probability of the training data given the model).

Since the Baum-Welch algorithm is a local iterative method, the resulting HMM and the number of required iterations depend heavily on the initial model. Of the many ways to generate an initial model, some techniques consider the training data while others do not.

The multiple observation training sequences are concatenated together to form one observation sequence for input into the Baum-Welch algorithm. All equations involved in the Baum-Welch process were obtained from Rabiner’s tutorial on HMMs In a nutshell, the re-estimated HMM parameters π_i_, a_ij_ and b_j_(k) are found using the following equations,

$${\overset{-}{\text{O}}}_{\text{i}}=\text{Expected}\;\text{frequency}\;(\text{number}\;\text{of}\;\text{times})\;\text{in}\;\text{state}{\text{N}}_{\text{i}}\;\text{at}\;\text{time}\;1$$

$${\text{a}}_{\text{ij}}=\frac{\text{expected number of transition from state i to state ij}}{\text{expected number of transition from state}\;{\text{N}}_{\text{i}}}$$

$${\text{b}}_{\text{j}}(\text{k})=\frac{\text{expected number of transition in state i and observing symbol k}}{\text{expected number of times in state i}}$$

## CONCLUSION

In this paper, a novel method of segmenting the CT images been discussed. This research work carried out by taking 4-D and 2-D CT images. Compared with 2-D Lung CT images 4-D images are Giving more detailed aspects of Lung details Unlike previous segmentation methods using ANN (9), the Lobe segmentation algorithm utilizes information from the bronchial tree without segmenting, so it reduces the computational time and complexity. The proposed work was carried out in 5 phases. In first phase, image acquisition of lung features and removal of the noise. Second phase is related to the segmentation of ROI features of lung which can be determined using segmentation algorithm such as region growing and Thresholding approach. Then the corresponding lung lobe is separated . Fourth phase is feature extraction, it extract the corresponding Lung nodule. Finally, the extracted lung nodule are classified. In this paper we analyses the result for 2-D and 4-D images. So early detection of Lung Cancer cells can be highly possible and it reduces the risk as well. This Bio-imaging methods will enhance the proper radiotherapy treatment for Lung Cancer patients.

## FUTURE SCOPE

By this process tie complexity is reduced and diagnosis confidence is increased. An clear identification of lungs Cancer whether it present or not by the CT images by using hidden Markov model. This process reduces the time complexity and increases the diagnosis confidence. The collected data contain noise the noises are removed. And then segmentation of the lung images and after that the image is separated. The lobes want to be separated and according to the features of the defected part we can conclude the cancer is present or not and the patient is in which stage. For calculation the output image is trained by using the HMM model and the diagnosis is made from the output.

Our current investigation is to further obtaining a clear identification of Lung Cancer for feature extraction and classification for 4-D CT image. We are trying to develop the automated medical image processing tools in which it detects the cancer cells in advance.
